# Multi-scale boron penetration toward stabilizing nickel-rich cathode

**DOI:** 10.1016/j.fmre.2022.03.001

**Published:** 2022-03-08

**Authors:** Bianzheng You, Zhixing Wang, Yijiao Chang, Wei Yin, Zhengwei Xu, Yuexi Zeng, Guochun Yan, Jiexi Wang

**Affiliations:** a School of Metallurgy and Environment, Central South University, Changsha 410083, China; b Energy Storage & Distributed Resources Division, Lawrence Berkeley National Laboratory, 1 Cyclotron Rd, Berkeley, CA 94720, USA; c Engineering Research Center of the Ministry of Education for Advanced Battery Materials, Central South University, Changsha 410083, China; d Hunan Provincial Key Laboratory of Nonferrous Value-Added Metallurgy, Central South University, Changsha 410083, China

**Keywords:** Nickel-rich layered oxides, Lithium borates, Intergranular cracks, Lithium-ion diffusion kinetics, Trace boron doping, Gas evolution

## Abstract

Nickel-rich layered oxides LiNi*_x_*Co*_y_*Mn_1-_*_x_*_-_*_y_*O_2_ (*x* ≥ 0.8) have been recognized as the preferred cathode materials to develop lithium-ion batteries with high energy density (>300 Wh kg^−1^). However, the poor cycling stability and rate capability stemming from intergranular cracks and sluggish kinetics hinder their commercialization. To address such issues, a multi-scale boron penetration strategy is designed and applied on the polycrystalline LiNi_0.83_Co_0.11_Mn_0.06_O_2_ particles that are pre-treated with pore construction. The lithium-ion conductive lithium borate in grain gaps functions as the grain binder that can bear the strain/stress from anisotropic contraction/expansion, and provides more pathways for lithium-ion diffusion. As a result, the intergranular cracks are ameliorated and the lithium-ion diffusion kinetics is improved. Moreover, the coating layer separates the sensitive cathode surface and electrolyte, helping to suppress the parasitic reactions and related gas evolution. In addition, the enhanced structural stability is acquired by strong B-O bonds with trace boron doping. As a result, the boron-modified sample with an optimized boron content of 0.5% (B5-NCM) exhibits a higher initial discharge capacity of 205.5 mAh g^−1^ at 0.1C (1C = 200 mA g^−1^) and improved capacity retention of 81.7% after 100 cycles at 1C. Furthermore, the rate performance is distinctly enhanced by high lithium-ion conductive LBO (175.6 mAh g^−1^ for B5-NCM and 154.6 mAh g^−1^ for B0-NCM at 5C).

## Highlights

• Multi-scale boron penetration from atomic to particle levels is proposed toward stabilizing Ni-rich cathode.
• Trace boron doping in TM sites of surface layer strengthens structural stability.
• Conductive LBO as binder facilitates Li^+^ diffusion and ameliorates intergranular cracks formation.
• LBO coated on primary/secondary particle surface alleviates gas evolution during electrochemical process.

## Graphical abstract

**Figure:**
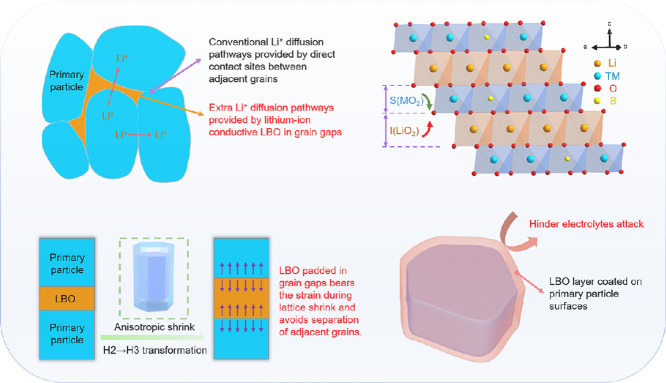

## 1 Introduction

With the abrupt development of electric vehicles (EVs), developing advanced cathode materials with high energy density is one of the main strategies to alleviate range anxiety among electric car drivers. Nickel-rich cathode materials LiNi*_x_*Co*_y_*Mn*_z_*O_2_ (*x* ≥ 0.8, NRCMs) with a capacity of more than 200 mAh g^−1^, attract much attention as promising candidates for the next-generation power batteries (>300 Wh kg^−1^). Nevertheless, the poor cycling stability and unsatisfied rate capability arise simultaneously with decreasing fraction of cobalt and manganese in NRCMs [1,2]. For the polycrystalline NRCMs, intergranular cracking is recognized as the dominant factor causing the deterioration of cycling performance, which roots in the repeated anisotropic shrinkage/expansion of lattices in the charge-discharge process due to the phase transformation between H2 and H3 [3,4]. Thereby, the tunnels for electrolyte penetration appear when microcracks spread to the surface of particles, causing severe interfacial reactions between primary particles and electrolytes that deteriorate the cycling performance of NRCMs. Another significant issue lies in the sluggish kinetics of lithium-ion diffusion, which is not able to support high-rate charge/discharge. It is because the interior lithium ions have to cross large amounts of grain boundaries to reach the surface of particles, and the inherent gaps between primary particles inhibit the diffusion [5]. As a result, a concentration gradient of lithium ions will be built in NRCM particles, where the surficial parts are in a deeper de-lithiation state during charging, causing accumulated internal stress and microcracks [6,7]. Hence, the poor kinetics of lithium-ion diffusion in NRCMs not only leads to the unsatisfied rate performance but also aggravates the formation of microcracks.

Many efforts have been made to tackle these aforementioned issues of NRCMs including elements doping [[8], [9], [10]], surface coating [[11], [12], [13]], morphology design [[14], [15], [16]], and gradient structure [17,18]. Coating is generally recognized as an effective strategy to ameliorate interfacial problems. Particularly, coating with high lithium-ion conductive matters is able to improve the lithium-ion diffusion kinetics and suppress the parasitic reactions simultaneously [19]. Nowadays, most of the coating strategies focus on the surface of secondary particles. However, NRCMs still suffer severe problems inside secondary particles. Consequently, it could be effective if the coating was conducted on the surface of primary particles inside secondary particles. For instance, Moonsu Yoon et al. [20] proposed a high-quality surface coating at both primary- and secondary-particle levels by cobalt boride metallic glass, and it significantly improved the cycling and rate properties of NRCMs.

**Fig. 1:**
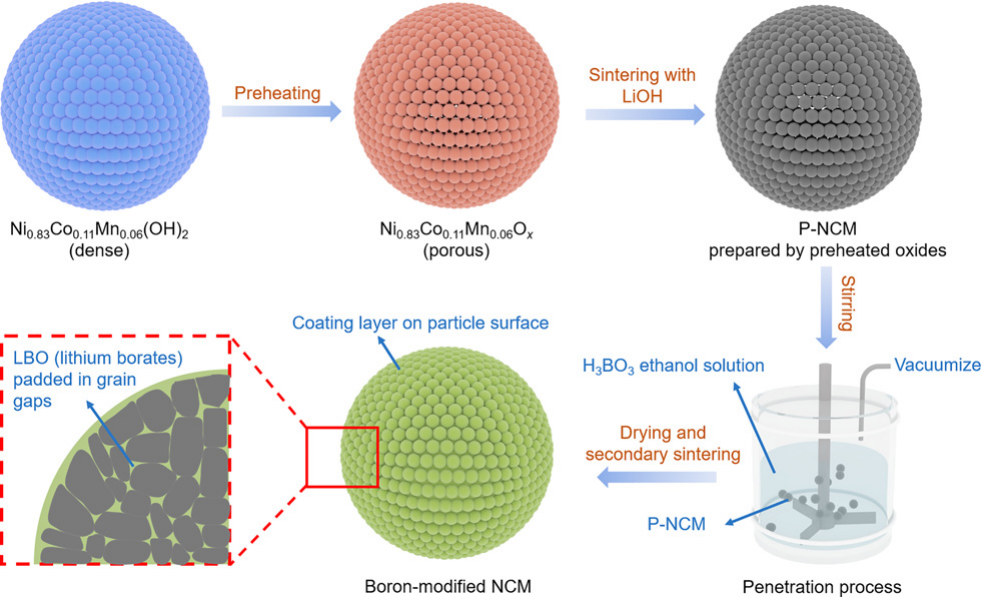
**Schematic diagram of the synthesis of LBO modified LiNi**_**0.83**_**Co**_**0.11**_**Mn**_**0.06**_**O**_**2**_**.**

Herein, a pore construction-boron penetration strategy is proposed to synthesize the lithium borates (LBO) coated LiNi_0.83_Co_0.11_Mn_0.06_O_2_ cathode materials, in which LBO is covered on the surface of both primary and secondary particles. By constructing pores inside the secondary particles by preheating Ni_0.83_Co_0.11_Mn_0.06_(OH)_2_ beforehand, the LiOH and H_3_BO_3_ are more ready enter into the particles as compared to the conventional solid-state synthetic methods (Fig. 1). This unique strategy is beneficial to forming the LBO coating layer onto the surface at both primary- and secondary-particle levels, suppressing the parasitic reactions between cathode and electrolyte, and alleviating the repeating formation and growth of cathode electrolyte interphase (CEI) film. Moreover, the lithium-ion-conductive LBO filled in the gaps between interior primary particles can prevent the formation and extending of intergranular cracks, and provide more diffusion pathways for lithium ions. Accordingly, both structural stability and lithium-ion-diffusion kinetics of LiNi_0.83_Co_0.11_Mn_0.06_O_2_ can be remarkably enhanced. Expectably, its cycling performance and rate capability can be improved. Putting such emphasis on the fundamental issues of NRCM materials, our work provides a feasible strategy to enhance their electrochemical performance.

## 2 Experimental section

### 2.1 Materials synthesis - NCM sample

To synthesize stoichiometric LiNi_0.83_Co_0.11_Mn_0.06_O_2_ sample (NCM), the Ni_0.83_Co_0.11_Mn_0.06_(OH)_2_ hydroxide precursor (provided by Hunan Shanshan Advanced Material Co. Ltd.) was mixed homogeneously with LiOH·H_2_O in a molar ratio of lithium to transition metal = 1.05. Then, the mixture was calcinated at 500 °C for 5 h followed by 780 °C for 15 h under O_2_ atmosphere in a quartz tube furnace with a heating rate of 5 °C min^−1^.

### 2.2 Materials synthesis - P-NCM sample

The Ni_0.83_Co_0.11_Mn_0.06_(OH)_2_ hydroxide precursor was heated at 600 °C for 3 h first to obtain Ni_0.83_Co_0.11_Mn_0.06_O*_x_* oxides, which was used as the precursor to prepare preheated Ni-rich material (P-NCM) by using the same synthetic protocol as the NCM sample.

### 2.3 Materials synthesis - boron-modified NRCMs

At first, 2 g of P-NCM were dispersed in 10 mL of ethanol. Then a certain amount of H_3_BO_3_ was added into the solution with continuous stirring for 20 min at room temperature. Afterward, 30 min of vacuuming was carried out to remove the gas in pores of cathode materials to facilitate the penetration of H_3_BO_3_ solution. After that, the suspension underwent a drying operation at 80 °C for 12 h. Eventually, the prepared H_3_BO_3_-cathode mixtures were calcined at 500 °C for 5 h to obtain the boron-modified NRCMs. On the basis of the molar ratio of boron to transition metals (0%, 0.1%, 0.5%, and 1%), the boron-modified NRCMs were denoted as B0-NCM, B1-NCM, B5-NCM, and B10-NCM in order. The samples were transferred into the glove box immediately after the secondary sintering to avoid contact with H_2_O and CO_2_ in air, making the residual lithium to be comprised of Li_2_O mainly rather than LiOH or Li_2_CO_3_.

### 2.4 Materials characterizations

N_2_ isothermal adsorption/desorption curves were measured at 77.3 K by Micromeritics ASAP 2020HD88. The corresponding specific surface area and pore width distribution were obtained from BET and BJH equations, respectively. The crystalline structure and lattice parameters were determined by XRD (Empyrean 2, PANalytical) using Cu Kα radiation operated at 40 kV in the 2θ range of 10°-80° with a scanning speed of 5° min^−1^. The valance states of the materials were determined by X-ray photoelectron spectroscopy (Thermo Scientific K-Alpha) with Al Kα radiation and all spectra were calibrated by C 1s (284.8 eV) as a reference. The morphology and the microstructure of the materials were determined by SEM (JSM-7900F, JEOL) and TEM (Titan G2, FEI). The samples for cross-section TEM observations were prepared by a focused ion beam (Helios Nanolab 600i, FEI). For XANES measurement, 2D images were taken at different energies across the Ni absorption edge (8.33–8.37 keV, 5 eV interval).

### 2.5 Electrochemical characterization

The electrochemical performance was tested by using a 2025 coin-type half-cell. The slurry containing 80 wt% of active materials, 10 wt% of carbon black (as the conductive agent), and 10 wt% of polyvinylidene fluoride (as the binder) in N-methylpyrrolidinone, was coated onto Al foil and dried in a vacuum oven at 90 °C for 6 h. Then, it was cut into discs with 12 mm in diameter with a typical mass loading of about 2.8 mg cm^−2^. The electrolyte was composed of LiPF_6_ (1 M) in a mixed solution of dimethyl carbonate (DMC), ethyl methyl carbonate (EMC), and ethylene carbonate (EC) (1:1:1 in volume ratio). The coin cells were assembled in an Ar-filled glove box. Electrochemical cycling tests were performed at 1C for 100 cycles (1C = 200 mA g^−1^) at 25 and 60 °C between 2.8 and 4.3 V. Before cycling tests, the cells were activated at 0.1C for 2-3 cycles. Rate capabilities were tested at 0.1C, 0.2C, 0.5C, 1C, 2C, 5C, 0.1C continuously with 5 cycles per rate. EIS was performed by Bio-Logic SP150 within the frequency range of 10^−2^–10^5^ Hz before cycling and after 100 cycles at lithiation state. Cyclic voltammetry (CV) was performed for the first 3 cycles between 2.8 and 4.3 V with a scan rate of 0.1 mV s^−1^. Galvanostatic intermittent titration techniques (GITT) were collected as follows. Firstly, the cells were charged with a constant charging current of 20 mA g^−1^ for 10 min. After that, the cells were relaxed for 50 min. The above two steps were repeated until the voltage of cells reached 4.3 V. Then the cells were discharged under the same condition with the cutoff voltage of 2.8 V.

## 3 Results and discussion

### 3.1 Pore-construction operation and influences

During the preheating process, the Ni_0.83_Co_0.11_Mn_0.06_(OH)_2_ hydroxide precursor turns into the Ni_0.83_Co_0.11_Mn_0.06_O_x_ oxide precursor. As increasing the heating temperature from 400 to 700 °C, the structure of the precursor transforms from Ni(OH)_2_ single phase to the coexistence of two phases (MnCo_2_O_4_ and NiO) (Fig. S1, detected by X-ray diffraction, XRD), and the pores are constructed because of the gas evolution and volume contraction of primary particles. However, big cracks appear when the temperature is higher than 700 °C (Fig. S2), which is not the desirable porousoxide precursor to synthesize LiNi_0.83_Co_0.11_Mn_0.06_O_2_. Thereby, the oxide precursor preheated at 600 °C, which balances the porous morphology and structural integrity, is utilized to prepare the cathode material (P-NCM) through high-temperature sintering. For comparison, the cathode material prepared by hydroxide precursor without preheating treatment is denoted as NCM.

**Fig. 2:**
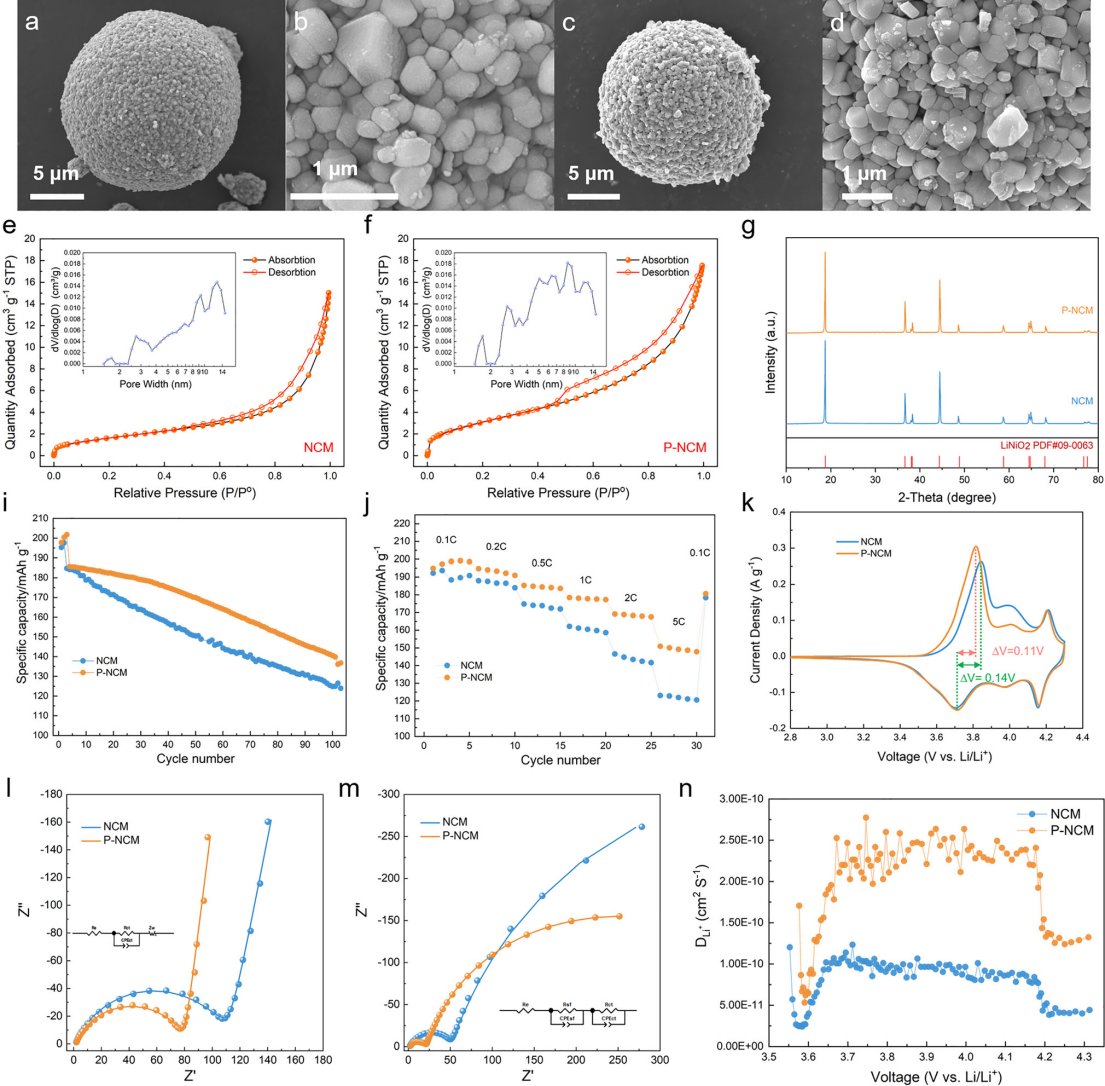
**Comparison in morphology, phase, specific surface area, pore volume, and electrochemical properties of NCM and P-NCM.** (a–d) SEM images of (a,b) NCM and (c,d) P-NCM; (e, f) N_2_ isothermal adsorption/desorption curves and pore size distributions; (g) XRD patterns; (i) Cycling performance with current density of 0.1C for first three cycles and 1C for next 100 cycles under 25 °C; (j) Rate capability at 0.1-5C under 25 °C; (k) CV curves with scan rate of 0.1 mV s^−1^ from 2.8 to 4.3 V; (l,m) EIS plots (l) before cycling and (m) after 100 cycles at 1C; (n) Diffusion coefficients of lithium ions measured by GITT.

The morphology, phase, specific surface area, pore volume, and electrochemical properties of NCM and P-NCM samples are compared in Fig. 2 to evaluate the influence of the pore-construction operation. As shown in Fig. 2a, b, NCM exhibits typical spherical secondary-particles assembled closely by nano-scaled polyhedral grains. On the contrary, small pores can be observed obviously between the external primary particles in P-NCM (Fig. 2c, d), arising from gas evolution during the preheating process. The N_2_ isothermal adsorption/desorption curves of NCM, P-NCM (Fig. 2e, f) and corresponding precursors (Fig. S3) are demonstrated to quantify the changes of a specific surface area and pore volume. The corresponding results are summarized in Table S1. The oxide precursor exhibits a much larger specific surface area than the hydroxide precursor, and the volume of pores ranging from 2 to 10 nm undergoes a significant increase after preheating operation (Fig. S3, Table S1), indicating that abundant pores have been successfully constructed inside secondary particles, which is consistent with the observation of SEM images. After the high-temperature sintering with lithium sources, NCM shows a slight increase in the specific surface area, pore volume, and average pore size compared with the hydroxide precursor (Fig. 2e, Table S1). A similar phenomenon can also be observed by comparing P-NCM and the oxide precursor (Fig. 2f, Table S1). Distinctly, the original pore structure constructed by the preheating operation is inherited in the subsequent high-temperature sintering process. Notably, the specific surface area and pore volume of NCM are still much lower than those of P-NCM, proving the necessity and advantage of preheating operation. The XRD patterns of NCM and P-NCM (Fig. 2g) reveal the typical hexagonal layered structure belonging to the space group of R-3m, indicating that the preheating operation does not affect the fundamental structure of the materials. The *I*_(003)_/*I*_(104)_ value is generally recognized as an indicator for the cation disordering degree [21]. Herein, the prepared samples exhibit similar ordering structures with the *I_(003)_/I_(104)_* values over 1.2 (Table S2), indicating a relatively low cation disordering degree. In addition, the Rietveld XRD refinements (Fig. S4, Table S2) show a typical layered structure of both NCM and P-NCM with the *c*/*a* values larger than 4.94. The electrochemical properties of NCM and P-NCM samples at room temperature (25 °C) are shown in Fig. 2i,j and Table S3. P-NCM and NCM separately exhibit an initial specific discharge capacity of 199.2 and 193.7 mAh g^−1^ at 0.1C (1 C = 200 mA g^−1^) from 2.8 to 4.3 V (versus Li^+^/Li). As performed at 1C after 100 cycles, P-NCM and NCM maintain 136.8 and 124.0 mAh g^−1^ with the capacity retention of 73.7% and 67.1%, respectively. Furthermore, P-NCM shows a remarkably elevated discharge capacity of 150.9 mAh g^−1^ at 5C (only 123.1 mAh g^−1^ for NCM). It can be found that the pore-construction operation improves the rate performance markedly, owing to the enlarged electrochemically-active area for Li^+^ insertion/extraction provided by internal pores. Generally, more active sites bring about aggravated parasitic reactions and lead to deteriorated cycling properties. However, P-NCM even exhibits a slightly improved cycling performance. This phenomenon needs further investigation. In the aspect of cyclic voltammetry (CV) results (Fig. 2k), the ∆V (the voltage difference between oxidation and reduction peaks) of P-NCM (0.11 V) is lower than that of NCM (0.14 V), indicating a lower polarization for P-NCM. The electrochemical impedance spectroscopy (EIS) was utilized to explore the electrochemical resistance evolution before cycling (Fig. 2l) and after 100 cycles (Fig. 2m). The corresponding values of resistances are summarized in Table S4. Specifically, the value of R_ct_ (charge transfer resistance) for NCM increases obviously from 110.7 to 897.1 Ω after 100 cycles at 1C while that of P-NCM only increases from 77.2 to 448.1 Ω. When it comes to the Li^+^ diffusion inside cathode materials measured by galvanostatic intermittent titration technique (GITT), the lithium-ion diffusion co-efficient (D_Li+_) of P-NCM is 2.43 times that of NCM (Figs. 2n and S5, Table S5). Hence, P-NCM exhibits better electrochemical kinetics in the aspect of charge transfer and lithium-ion diffusion as compared with NCM, and this contributes to the improved electrochemical performance.

### 3.2 Penetrating LBO into grain boundaries and gaps of NRCMs

Boron-modified NRCMs were prepared from P-NCM through the penetration-drying-sintering routine (Fig. 1). Firstly, P-NCM was added into H_3_BO_3_-dissolved ethanol solution with continuous stirring in the vacuum environment. In this process, the residual lithium and H_3_BO_3_ penetrated secondary particles along the tunnels that were constructed by micropores. Afterward, the residual lithium and H_3_BO_3_ were deposited evenly on the particle surfaces and grain gaps by low-temperature evaporation at 60 °C with continuous stirring. The modified materials were eventually obtained in the secondary sintering process when LBO arose through the reaction between the residual lithium and pre-decomposed H_3_BO_3_ (Fig. S6). The boron-modified NRCMs marked as B0-NCM, B1-NCM, B5-NCM and B10-NCM represent the samples with boron to transition metal molar ratio of 0%, 0.1%, 0.5%, and 1%, respectively.

**Fig. 3:**
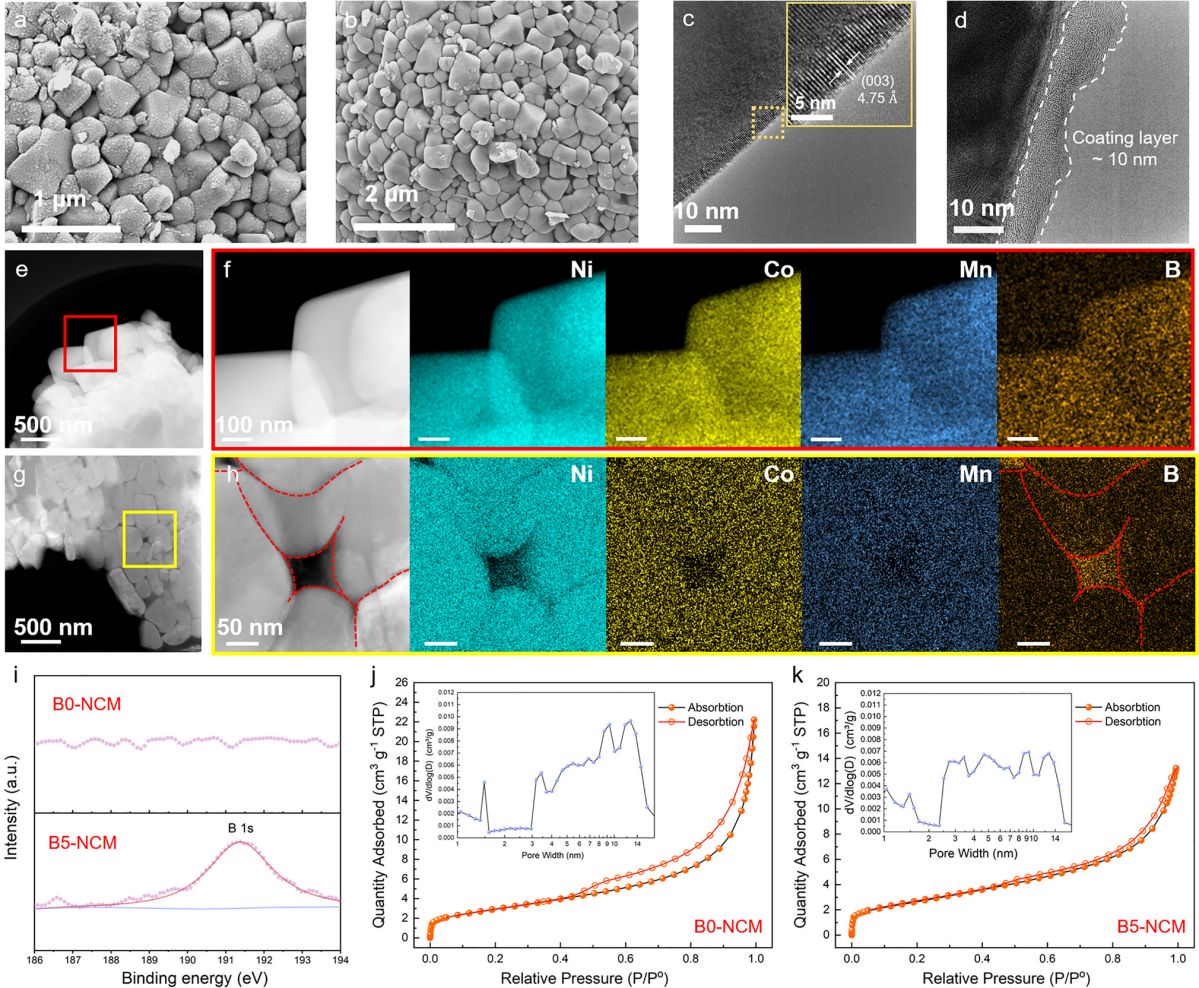
**Verification of uniform distribution of boron on both primary and secondary particle surfaces, and in gaps between primary particles.** (a, b) SEM images of (a) B0-NCM and (b) B5-NCM; HRTEM images of (c) B0-NCM and (d) B5-NCM; HAADF-STEM images of (e) B5-NCM and (f) corresponding EDS mapping; Cross-sectional HAADF-STEM images of (g) B5-NCM and (h) corresponding EDS mapping; (i) XPS spectra of B 1s for B0-NCM and B5-NCM; (j, k) N_2_ isothermal adsorption/desorption curves and pore size distributions (inset) of (j) B0-NCM and (k) B5-NCM.

The morphologies of boron-modified NRCMs are shown in Figs. 3a, b and S7. After the penetration-drying-sintering treatment, a large amount of residual lithium arises on the surface of B0-NCM particles (Fig. 3a), resulting from the dissolution of surficial lithium in ethanol solution and the subsequent deposition on the surface in the drying process. Nevertheless, this phenomenon disappears in the boron-modified samples (Fig. 3b, B5-NCM serves as the representative for boron-modified materials herein), revealing that the residual lithium has been consumed via the reaction with boron oxide in the secondary sintering process. Besides, the decrease in the pH value was observed after sintering in our previous work [19], confirming the consumption of residual lithium by H_3_BO_3_. Notably, the coating layer can be distinctly observed in the boron-modified samples, while the agglomeration of coating layer occurs in B10-NCM as increasing the addition of boron (Fig. S7). The high-resolution transmission electron microscopy (HRTEM) image of B0-NCM (Fig. 3c) shows a clean surface without coating layer. The clear lattice fringes separated by 4.75 Å correspond to (003) plane of the hexagonal layered structure. On the contrary, an amorphous layer of nearly 10 nm in thickness coats on the surface of B5-NCM (Fig. 3d). The EDS mapping results (Fig. 3e, f) confirm the homogeneous distribution of Ni, Co, Mn, and B on the surface of secondary particles. Furthermore, in order to investigate the interior of secondary particles, the cross-section diagrams and element distributions of B5-NCM analyzed by scanning TEM-energy dispersive X-ray spectroscopy (STEM-EDS) are illustrated in Figs. 3g, h and S8. The grain boundaries and gaps between the primary particles ranging from 50 to 200 nm can be observed clearly. The strong signals of B are acquired in different regions, suggesting that B penetrates from the surface of particles to interior along the tunnels and fills the grain gaps. In addition, a strong signal of B 1s is acquired in B5-NCM from XPS spectra but not in B0-NCM (Fig. 3i), verifying the existence of boron on the surface of B5-NCM particles after boron addition. Furthermore, B5-NCM exhibits a smaller pore volume and average pore size as compared with B0-NCM. Meanwhile, the difference of median value of micropore width between B0-NCM and B5-NCM is negligible (Fig. 3j, k, Table S1), indicating that the B prefers to penetrate and pad larger pores rather than micropores inside the secondary particles. The above results demonstrate that the boron-contained chemical is evenly coated on the surface of both secondary particles and interior primary grains, and fills the grain gaps successfully by a penetration-drying-sintering strategy.

### 3.3 Enhanced electrochemical performance and mechanism analysis

**Fig. 4:**
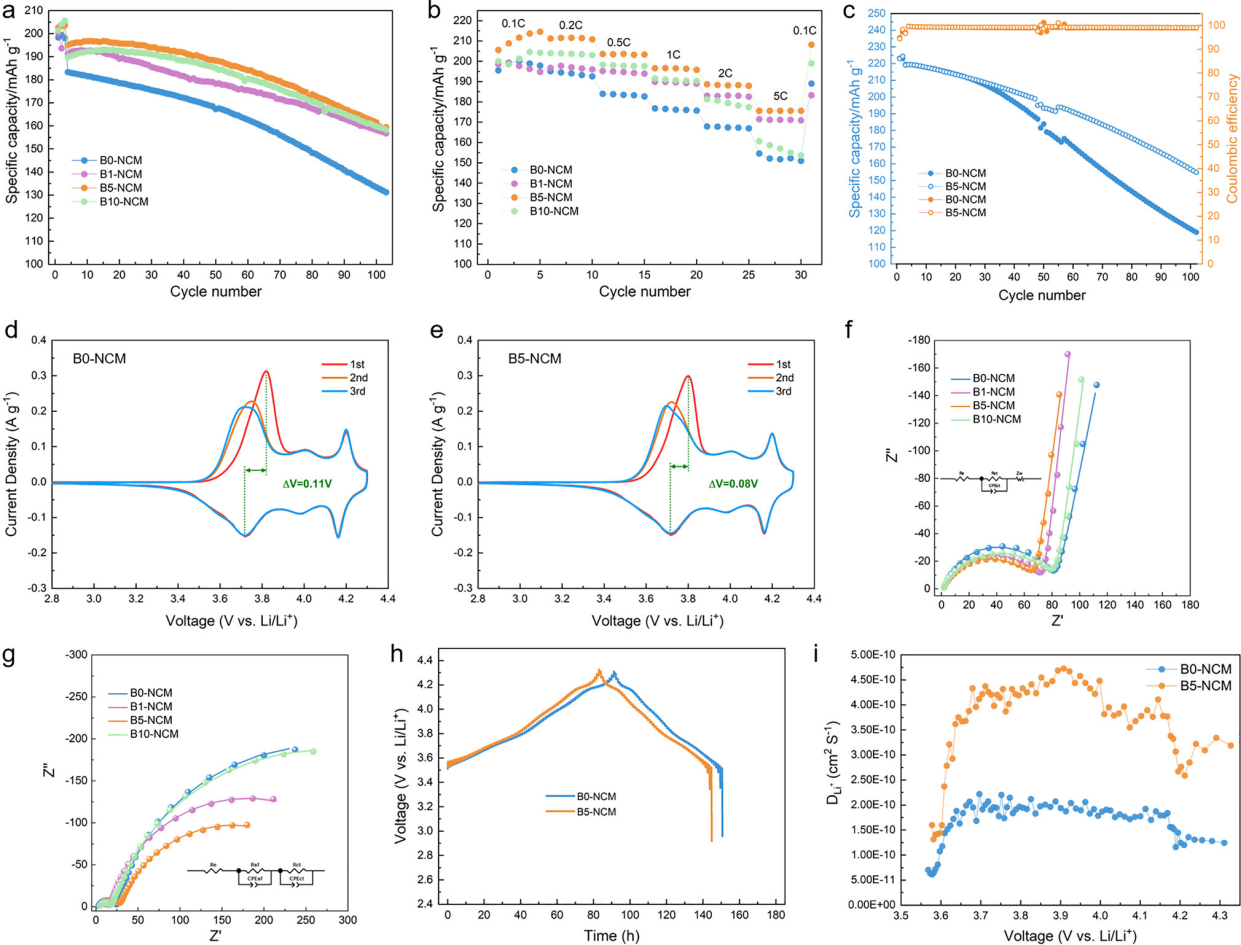
**Electrochemical performance of boron-modified NRCMs.** (a) Cycling performance with a current density of 0.1C for the first three cycles and 1C for the next 100 cycles at 25 °C; (b) Rate properties with different current densities of 0.1–5C at 25 °C; (c) Cycling performance of B0-NCM and B5-NCM with a current density of 0.1C for first two cycles and 1C for next 100 cycles at 60 °C; (d, e) CV curves of (d) B0-NCM and (e) B5-NCM with a scanning rate of 0.1 mV s^−1^ from 2.8 to 4.3 V; (f, g) EIS Nyquist diagrams and fitted curves for boron-modified NRCMs (f) before cycling and (g) after 100 cycles; insert: equivalent circuits. (h) GITT curves and (i) corresponding lithium-ion diffusion coefficients of B0-NCM and B5-NCM.

The systematic electrochemical testing for the prepared cathode materials is implemented at 25 and 60 °C to thoroughly evaluate the influence of LBO modification. The comparison of cycling and rate performance at 25 °C for the boron-modified NRCMs are summarized in Fig. 4a, b and Table S3. B0-NCM exhibits a specific discharge capacity of 200.2 mAh g^−1^ in the first cycle at 0.1C (1C = 200 mA g^−1^) from 2.8 to 4.3 V (versus Li^+^/Li). After 100 cycles at 1C, it delivers a reversible capacity of 134.7 mAh g^−1^ with a capacity retention of 73.5%. Both specific capacity and capacity retention are similar to that of P-NCM, indicating the penetration-drying-sintering operation without boron addition makes negligible influence on the electrochemical performance. For the boron-modified materials, the initial specific discharge capacities at 0.1C are 199.2, 205.5, 204.6 mAh g^−1^, and the capacity retention after 100 cycles at 1C is 82.0%, 81.7%, 83.4% for B1-NCM, B5-NCM, B10-NCM, respectively (Fig. 4a). Distinctly, the initial discharge capacity and cycling stability are both enhanced by boron modification, and the electrochemical properties exhibit a close association with the content of boron addition. Particularly, the specific discharge capacity increases obviously with the increase in the content of boron from 0.1% to 0.5%, but the capacity retention shows an opposite trend. When it reaches 1%, the specific capacity undergoes a slight decrease while the capacity retention is somewhat improved. It is because the electrochemically-isolated boron oxides shall appear on the surface of particles when the residual lithium is used up with the increase in the content of boron, decreasing the active mass ratio while constructing a stable surface. Besides, as presented in Fig. 4b, the capacities at the high current density of 5C are 154.6, 171.5, 175.6, and 160.6 mAh g^−1^ for B0-NCM, B1-NCM, B5-NCM, and B10-NCM, respectively. The rate capability is significantly improved with boron addition, and B5-NCM shows the highest capacity at every current density. When the content of boron reaches 1%, the isolated layer hinders the diffusion of lithium ions between the cathode surface and electrolyte, resulting in a relatively poor rate capability for B10-NCM. Hence, B5-NCM exhibits the best electrochemical performance under a comprehensive consideration of initial discharge capacity, cycling stability and rate capability. Fig. 4c and Table S6 present the cycle performance of B0-NCM and B5-NCM at 60 °C. Before being cycled at 1C for 100 cycles, the cells were activated at 0.1C for the first two cycles. No matter at 0.1C or 1C, B0-NCM shows almost the same discharge capacity as that of B5-NCM at 60 °C. Nevertheless, the capacity retention of B5-NCM (70.6%) after 100 cycles is much higher than that of B0-NCM (54.2%), exhibiting an increased gap as compared with the room-temperature performance. The results demonstrate that the boron modification improves the cycling stability of NRCMs significantly at both room and elevated temperatures. The ∆V between the redox peaks of B5-NCM (0.09 V) in CV curves is lower than that of B0-NCM (0.11 V) (Fig. 4d, e), suggesting that B5-NCM exhibits a lower polarization [22] in the electrochemical process. Furthermore, the results of electrochemical resistance (Fig. 4f, g, Table S4) reveal that B5-NCM owns the lowest R_ct_ of 56.9 Ω (68.5 Ω for B0-NCM, 65.1 Ω for B1-NCM and 72.0 Ω for B10-NCM) before cycling and 268.0 Ω (483.9 Ω for B0-NCM, 325.9 Ω for B1-NCM and 503.5 Ω for B10-NCM) after 100 cycles. The boron modification successfully decreases the charge-transfer resistance. However, excessive boron in B10-NCM causes the generation of electrochemically-isolated layer on the surface of particles and thereby hinders the charge transfer across it. Taking the electrochemical performance and aforementioned BET surface area results into consideration, B5-NCM exhibits an obvious enhancement in rate performance although the specific surface area decreases, indicating that the amount of electrochemically-active area is not responsible for the improvement in the rate property of boron-modified materials. Hence, GITT measurements (Fig. 4h,i, Table S5) are utilized to further investigate the lithium-ion diffusion kinetics. The average D_Li+_ of B5-NCM is 2.17 times that of B0-NCM (1.69 × 10^−10^ cm^2^ S^−1^ for B0-NCM and 3.66 × 10^−10^ cm^2^ S^−1^ for B5-NCM), suggesting that the lithium-ion diffusion kinetics is improved after boron addition. Given the decrease of D_Li_^+^ for B0-NCM compared with P-NCM, the penetration-drying-sintering operation does not make positive effects on the lithium-ion diffusion and even makes deteriorations. Therefore, the improvement in lithium-ion diffusion kinetics of B5-NCM directly originates from the boron penetration. Specifically, the fast lithium-ion conductor LBO filled in the gaps between primary particles provides more pathways for lithium-ion diffusion at the grain boudaries. As a result, the electrochemical polarization is reduced These should be responsible for the superior rate property of boron-modified samples.

**Fig. 5:**
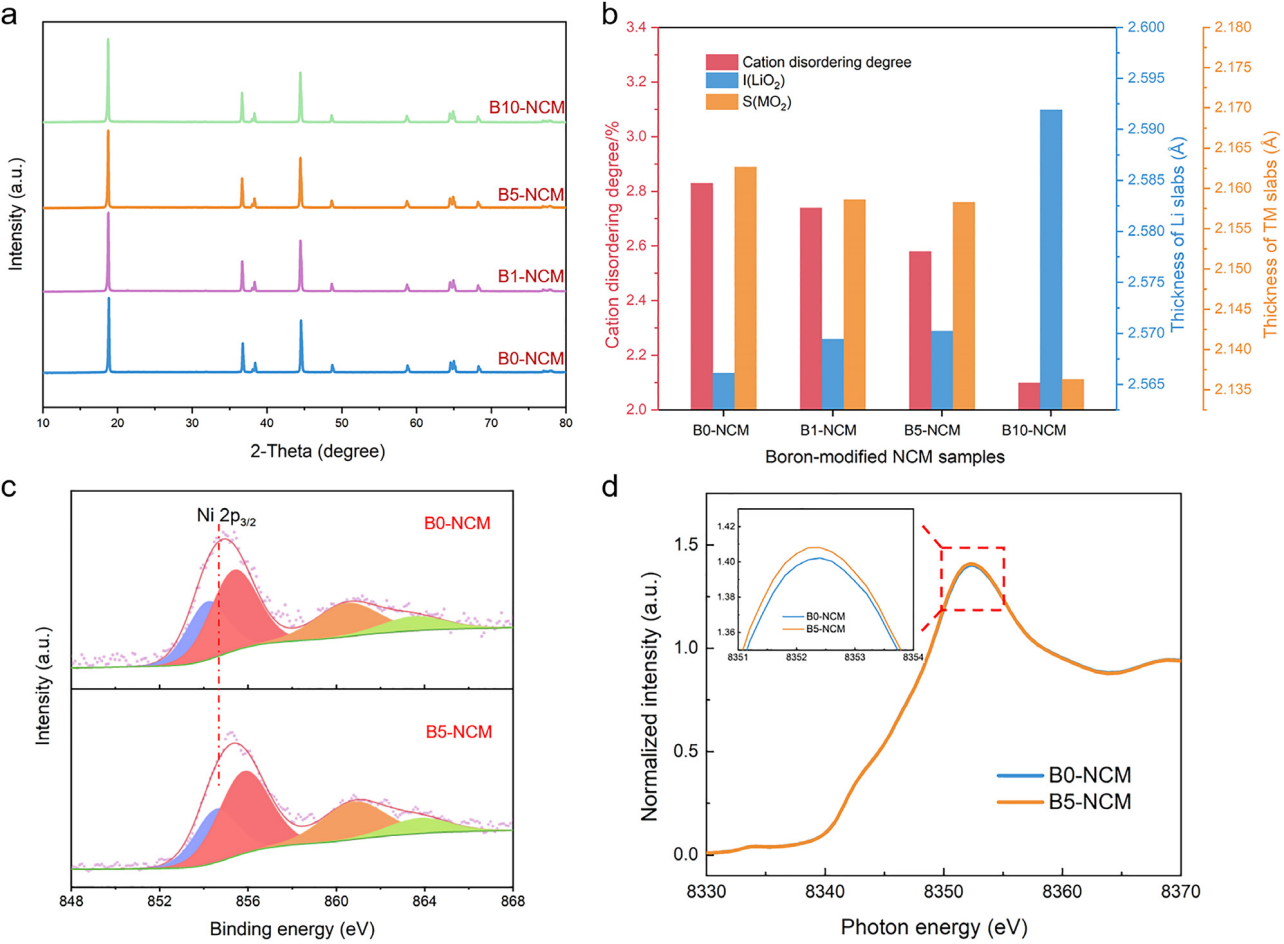
**Phase and valance evolutions after boron modification.** (a) X-ray diffraction patterns of B0-NCM, B1-NCM, B5-NCM, and B10-NCM; (b) relation between boron contents, cation disordering degree, I(LiO_2_) and S(MO_2_); (c) XPS spectra of Ni 2p_3/2_ for B0-NCM and B5-NCM; (d) Ni K-edge XANES spectra for B0-NCM and B5-NCM.

The XRD patterns of boron-modified NRCMs are shown in Fig. 5a. All samples exhibit a typical hexagonal layered structure belonging to the space group of R-3m, indicating that the addition of boron does not damage the fundamental structure of materials. They all show a perfect layered structure owing to the splits of (006)/(012) and (018)/(110) peaks [23]. To further investigate the influence of boron penetration on the lattice parameters, the results of Rietveld XRD refinement are presented in Fig. S9 and Table S2. The degree of cation disordering undergoes a nonnegligible decrease as increasing the amount of boron and B10-NCM exhibits the lowest value of 2.10% (2.83%, 2.74% and 2.58% for B0-NCM, B1-NCM and B5-NCM, respectively) (Fig. 5b, Table S2). The amelioration of cation disordering provides more active sites for lithium-ion insertion/extraction, reduces the resistance for lithium-ion diffusion and eventually contributes to the improvement in electrochemical performance. Besides, the value of I(LiO_2_) (thickness of Li slab) increases obviously with boron addition while that of S(MO_2_) (thickness of transition metal slab) shows an opposite tendency, indicating that boron may occupy the transition metal (TM) sites rather than the Li sites. This is because the bonds of B-O own a smaller bond length compared with those of TM-O and Li-O, making oxygen atoms move closer to boron by the strong attraction. Besides, the boron atoms in TM sites are able to ameliorate the generation of oxygen vacancy and enhance the structural stability by the strong B-O bonds [24]. Furthermore, the enlarged Li slabs contribute to the improvement in lithium-ion diffusion [25]. As shown in Fig. 5c, a distinct shift of Ni 2P_3/2_ peak to higher binding energy is observed with boron addition. This result reveals that the trace boron doping elevates the average valance of nickel near the surface of particles (i.e., the proportion of Ni^3+^ increases), resulting in the amelioration of cation disordering which is consistent with the results of Rietveld refinement. X-ray absorption near edge structure (XANES) was employed to further evaluate the influence of boron on the structure of the bulk materials. As shown in Fig. 5d, the XANES curves of Ni exhibit negligible variation as increasing the content of boron from 0 to 0.5%, indicating the structure maintains well after boron modification. Furthermore, the peak position of B5-NCM is almost identical to that of B0-NCM, which is quite different from the result of XPS. This phenomenon suggests that trace boron doping does not impact the average Ni valance of the whole materials distinctly. In summary, trace boron dopes into the surface lattice and elevates the average valance of Ni near the surface of particles, resulting in the amelioration of cation disordering. Besides, strong attraction between B and O atoms induces the contraction of TM slabs and expansion of Li slabs, contributing to the enhancement of structural stability and acceleration of lithium-ion diffusion.

**Fig. 6:**
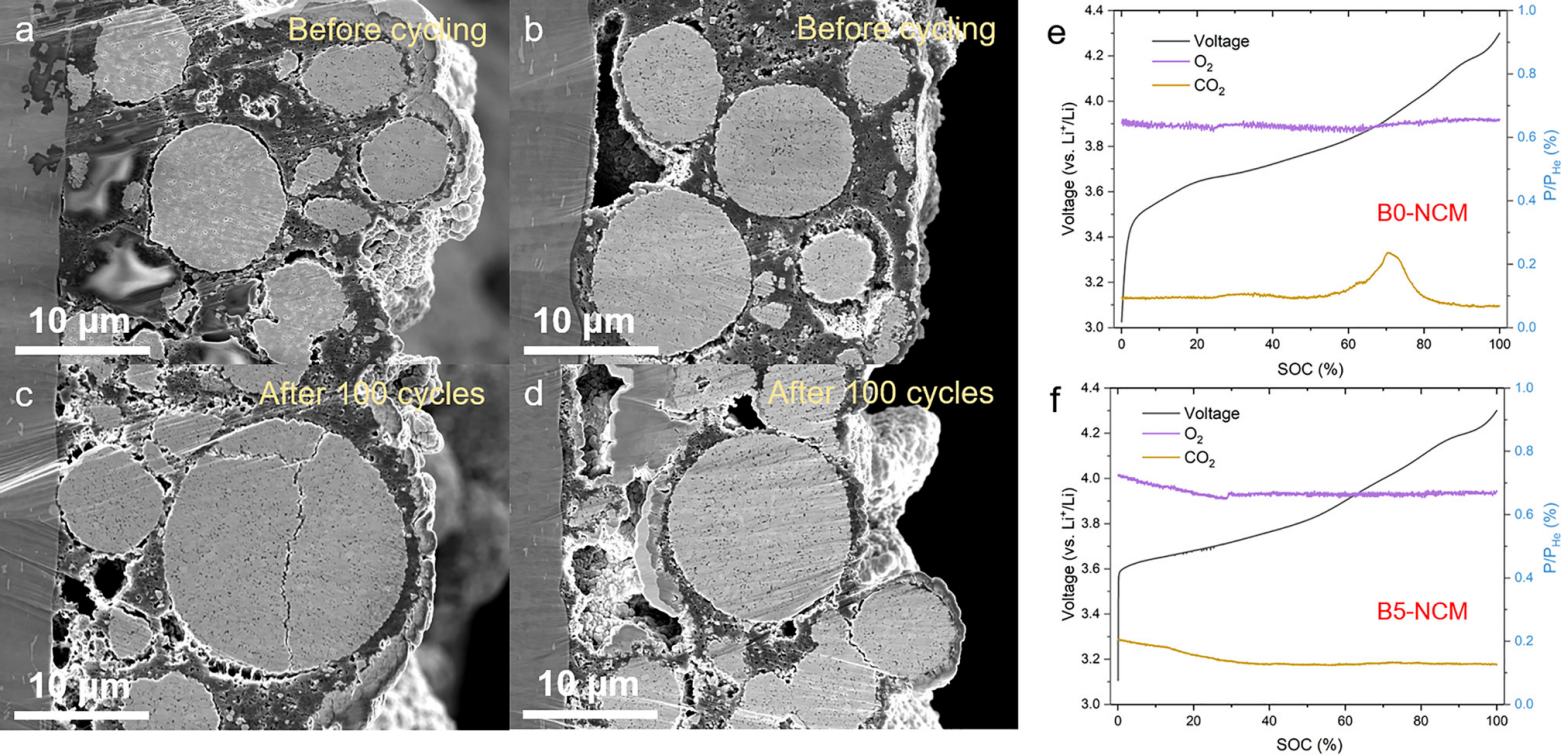
**Intergranular cracks and gas evolution of B0-NCM and B5-NCM during the electrochemical process.** (a–d) Cross-section SEM images of (a, c) B0-NCM and (b, d) B5-NCM (a, b) before cycling and (c, d) after 100 cycles with a current density of 1 C in the voltage of 2.8–4.3 V at 25 °C. (e, f) *In situ* DEMS data of (e) B0-NCM and (f) B5-NCM during first charge at 0.1C in the voltage range 3.0–4.3 V at 25 °C.

Microcracks are generally recognized as a dominated factor of structural stability and electrochemical properties [26]. Fig. 6 shows the cross-section SEM images of B0-NCM and B5-NCM before cycling and after 100 cycles. Both B0-NCM (Fig. 6a) and B5-NCM (Fig. 6b) samples before cycling show an integrated and dense morphology without any microcrack. After 100 cycles, distinct intergranular cracks across the whole secondary particle are observed in B0-NCM (Fig. 6c), whereas B5-NCM shows negligible crack and inherits the original morphology (Fig. 6d), demonstrating the mechanical enhancement of B5-NCM induced by boron penetration. Particularly, the LBO padded in grain gaps is able to bear and disperse the strain from adjacent grains which undergo an anisotropic contraction during H2→H3 phase transformation in the charging process. As such, the strain concentration and subsequent separation of contacted grains are avoided. This function is also effective in the discharging process when the anisotropic expansion occurs. Free of intergranular cracks in the boron-modified samples makes dominant influences on improving the cycling stability. Besides, the results of in-situ differential electrochemical mass spectrometry (DEMS) reveal that much less gas evolution of CO_2_ and O_2_ is detected during the first charging process for B5-NCM compared with B0-NCM (Fig. 6g, h), indicating the oxidation of electrolyte is suppressed remarkably. It demonstrates that the LBO layer coated on the grain surface inside secondary particles avoids direct contact between the electrolyte and cathode material, resulting in the amelioration of parasitic reactions. Furthermore, the structural enhancement arising from trace boron doping also contributes to the suppression of oxygen evolution [20,27]. Hence, the improvement in cycling stability originates from the structural stability at both atomic and micron levels, and from the suppression of parasitic reactions.

**Fig. 7:**
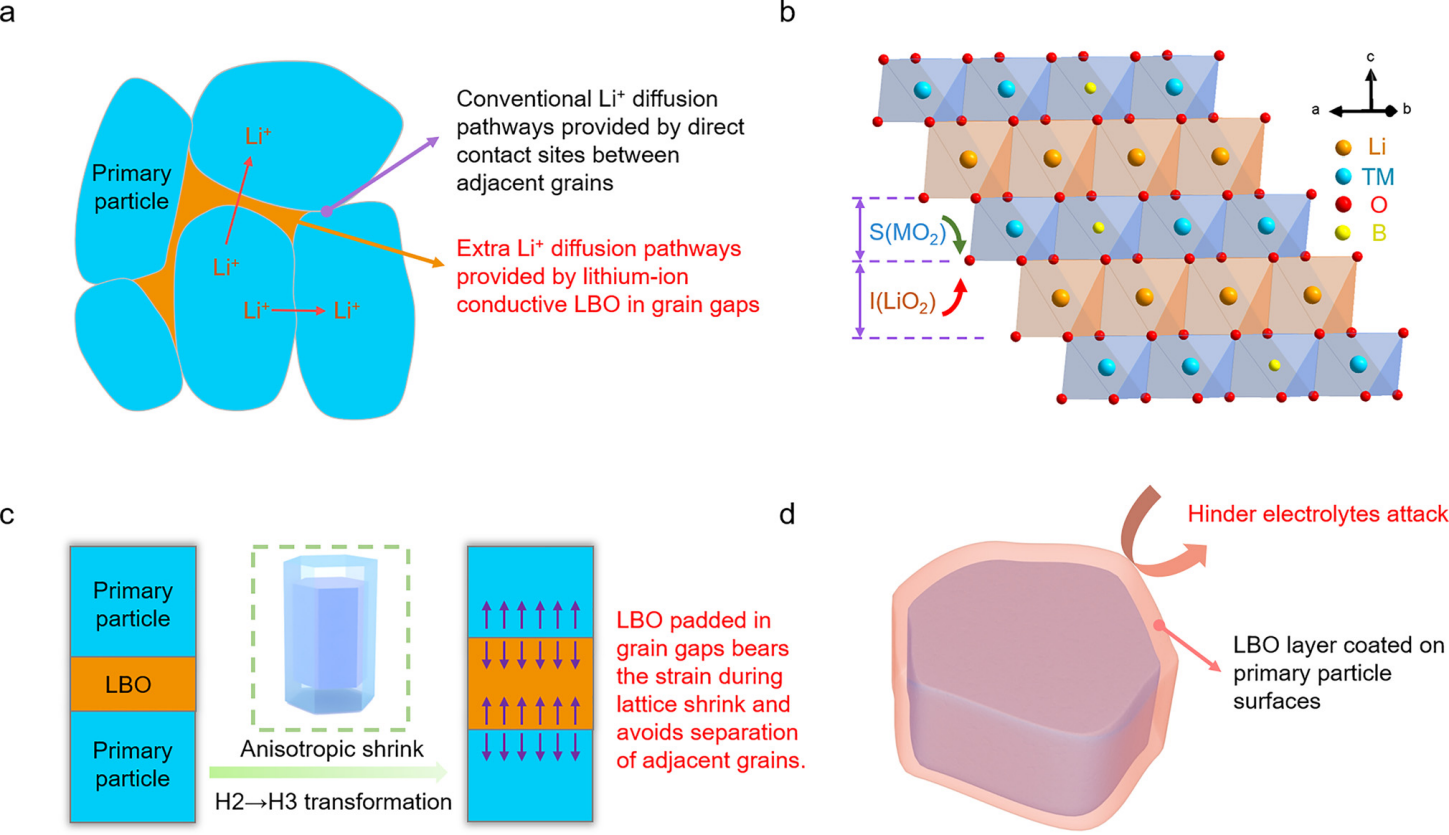
**Mechanism of property enhancement for boron-modified NRCM materials through pore construction- boron penetration strategy.** Note that I(LiO_2_) and S(MO_2_) represent the thickness of Li slab and transition metal slab, respectively.

Based on the above discussion, the mechanism diagram for improving the performance of NRCMs by pore construction-boron penetration strategy is illustrated in Fig. 7. Firstly, the LBO with high lithium-ion conductivity padded in grain boundaries and gaps provides more pathways and reduces the distance for lithium ions diffusion (Fig. 7a), significantly improving the Li^+^ diffusion kinetics and rate performance. Secondly, the trace boron doping on the surface and subsurface of grains enhances the structural stability via the strong B-O bonds (Fig. 7b). Specifically, the increased distance of lithium slabs contributes to the acceleration of lithium-ion diffusion. Thirdly, the LBO padded in grain gaps functions as a binder for adjacent grains which bears the strain in the anisotropic shrinkage process and stress in the anisotropic expansion process, avoiding strain concentration and subsequent intergranular cracks generation (Fig. 7c). Finally, the coating layer avoids direct contact between the sensitive cathode surfaces and electrolytes, distinctly ameliorating the parasitic reactions and suppressing the related gas evolution (Fig. 7d).

## 4 Conclusion

Boron-modified NRCM materials have been successfully prepared through a feasible pore construction-boron penetration strategy. The corresponding advantages of as-prepared materials were summarized as follows: (a) LBO with high lithium-ion conductivity padded in grain gaps significantly improves the Li^+^ diffusion kinetics. (b) Trace boron doping on the surface enhances the structural stability and increases the distance of lithium slabs, contributing to the acceleration of lithium-ion diffusion. (c) The LBO functions as a binder of adjacent grains to avoid the intergranular cracks generation. (d) The coating layer avoids the direct contact between cathode and electrolyte, ameliorating the parasitic reactions and suppressing the related gas evolution. Originated from the above-mentioned structural stability at both atomic and micron levels, accelerated lithium-ion diffusion between grains and suppressed parasitic reactions at electrode/electrolyte interfaces, both cycling and rate properties of the boron-modified NRCM materials were significantly enhanced. This article provides a novel way to improve the electrochemical properties of nickel-rich NRCM materials that can be employed for next-generation LIBs.

## Declaration of competing interest

The authors declare that they have no conflicts of interest in this work.

## Appendix Supplementary materials

Image, application 1
