# Supplementary material for: Multi-scale boron penetration toward stabilizing nickel-rich cathode
Source: Fundam Res. 2022 Mar 8;3(4):618–26. doi: 10.1016/j.fmre.2022.03.001 (PMC11197729; doi:10.1016/j.fmre.2022.03.001)
Supplement: Supplementary file 1 [file mmc1.docx]

**Supporting Information**

**Multi-scale boron penetration toward stabilizing nickel-rich cathode**

Bianzheng You,^a^ Zhixing Wang,^a,c,d^ Yijiao Chang,^a^ Wei Yin,^b^ Zhengwei Xu,^a^ Yuexi Zeng,^a^ Guochun Yan,^a,c,d,*^ Jiexi Wang^a,c,d,*^

^a^ School of Metallurgy and Environment, Central South University, Changsha 410083, P. R. China

^b^ Energy Storage & Distributed Resources Division, Lawrence Berkeley National Laboratory, 1 Cyclotron Rd, Berkeley, CA 94720, USA

^c^ Engineering Research Center of the Ministry of Education for Advanced Battery Materials, Central South University, Changsha, 410083, P. R. China

^d^ Hunan Provincial Key Laboratory of Nonferrous Value-Added Metallurgy, Central South University, Changsha, 410083, P. R. China

* Email address: happyygc@csu.edu.cn (G. Yan); wangjiexikeen@csu.edu.cn (J. Wang)


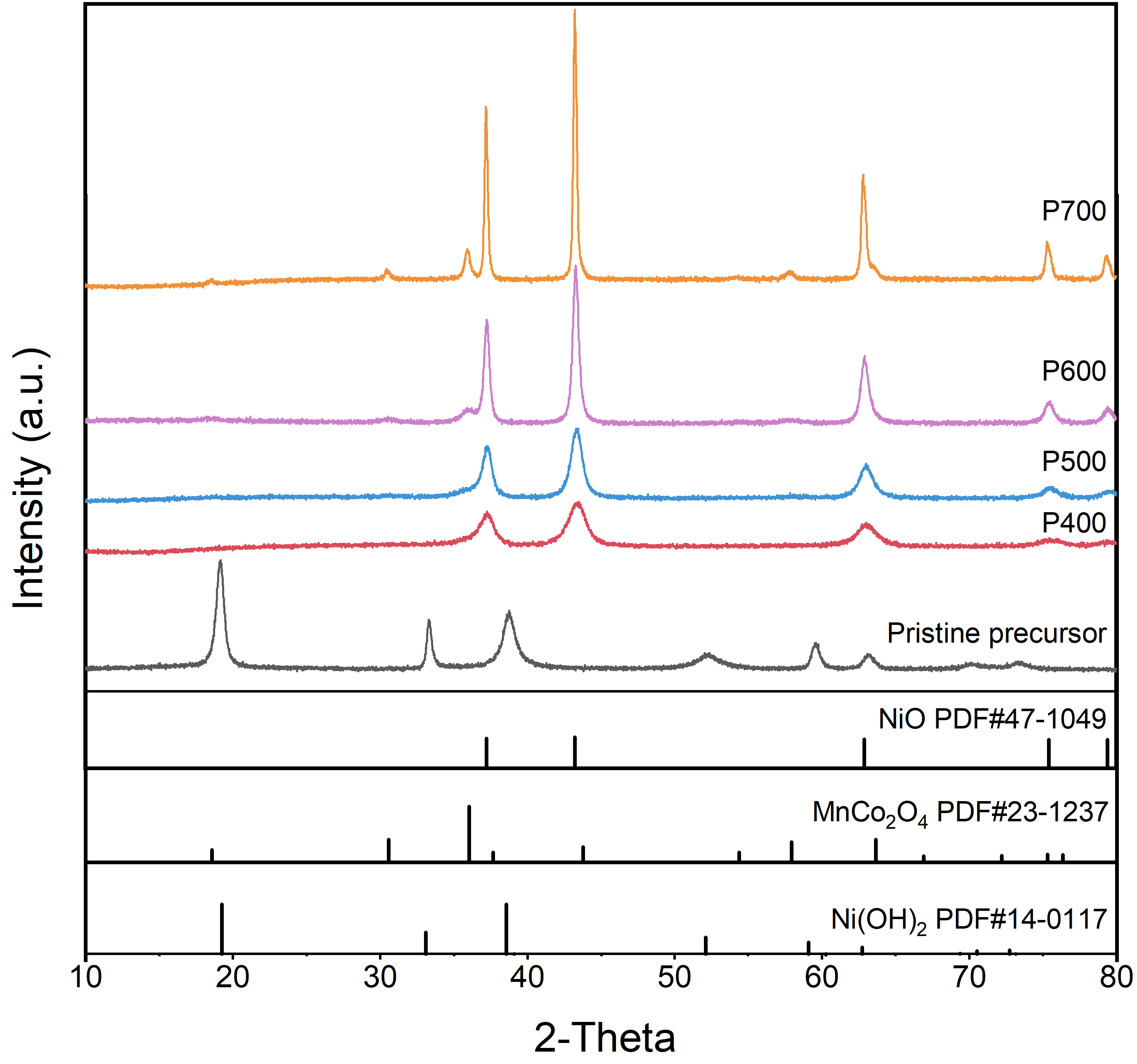


Figure S1. XRD patterns of Ni_0.83_Co_0.11_Mn_0.06_(OH)_2_ precursors before and after preheating operation.


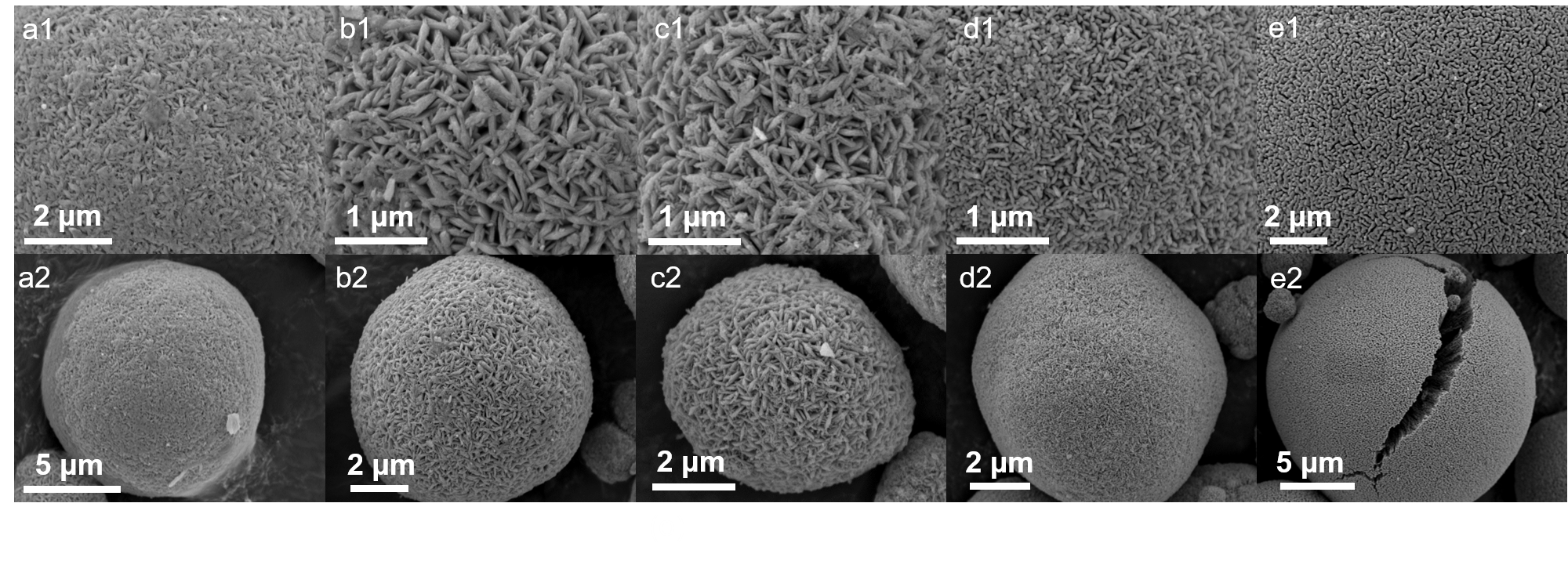


Figure S2. Morphologies of preheated precursors: (a1-a2) Pristine Ni_0.83_Co_0.11_Mn_0.06_(OH)_2_ precursor; (b1-b2) 400 ^o^C; (c1-c2) 500 ^o^C; (d1-d2) 600 ^o^C; (e1-e2) 700 ^o^C;


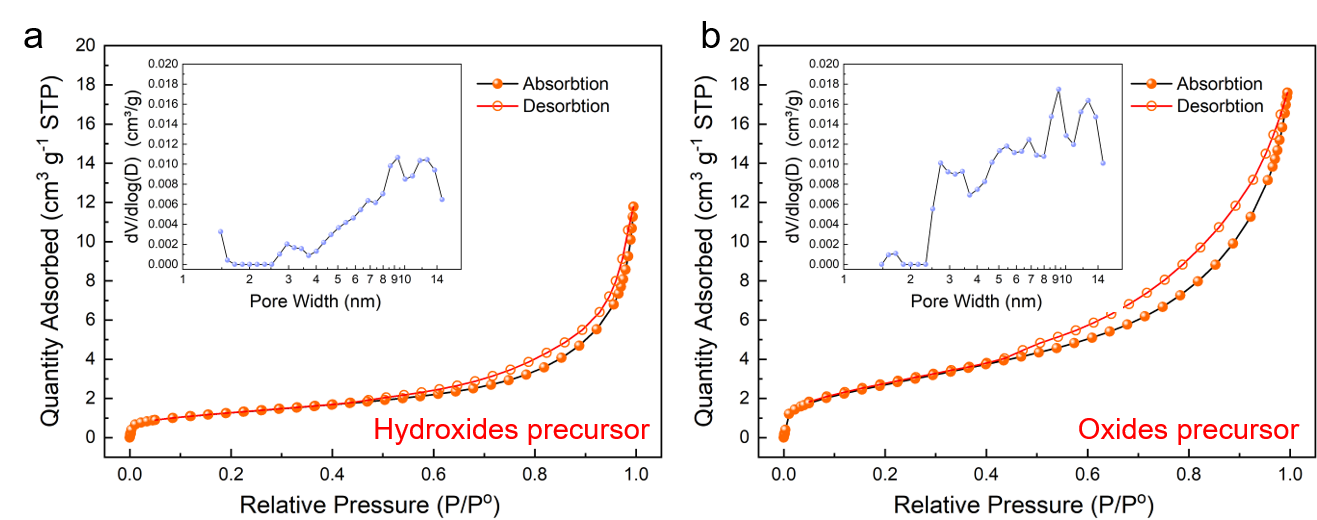


Figure S3. BET curves and pore width distribution (insert) of (a) hydroxide precursor Ni_0.83_Co_0.11_Mn_0.06_(OH)_2_ and (b) oxide precursor Ni_0.83_Co_0.11_Mn_0.06_O_x_.


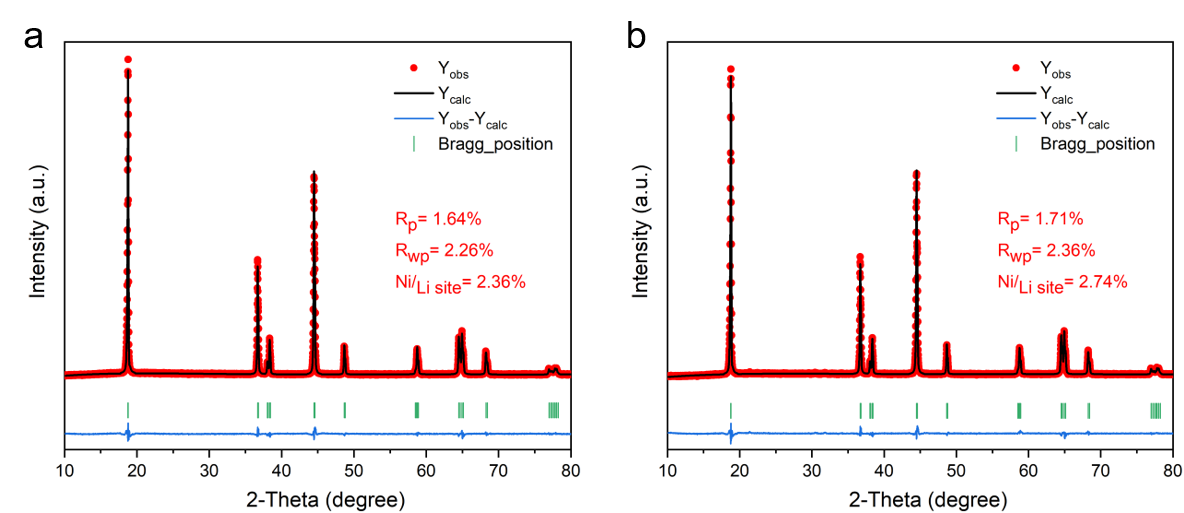


Figure S4. Rietveld refinement results of X-ray diffraction patterns for (a) NCM; (b) P-NCM.


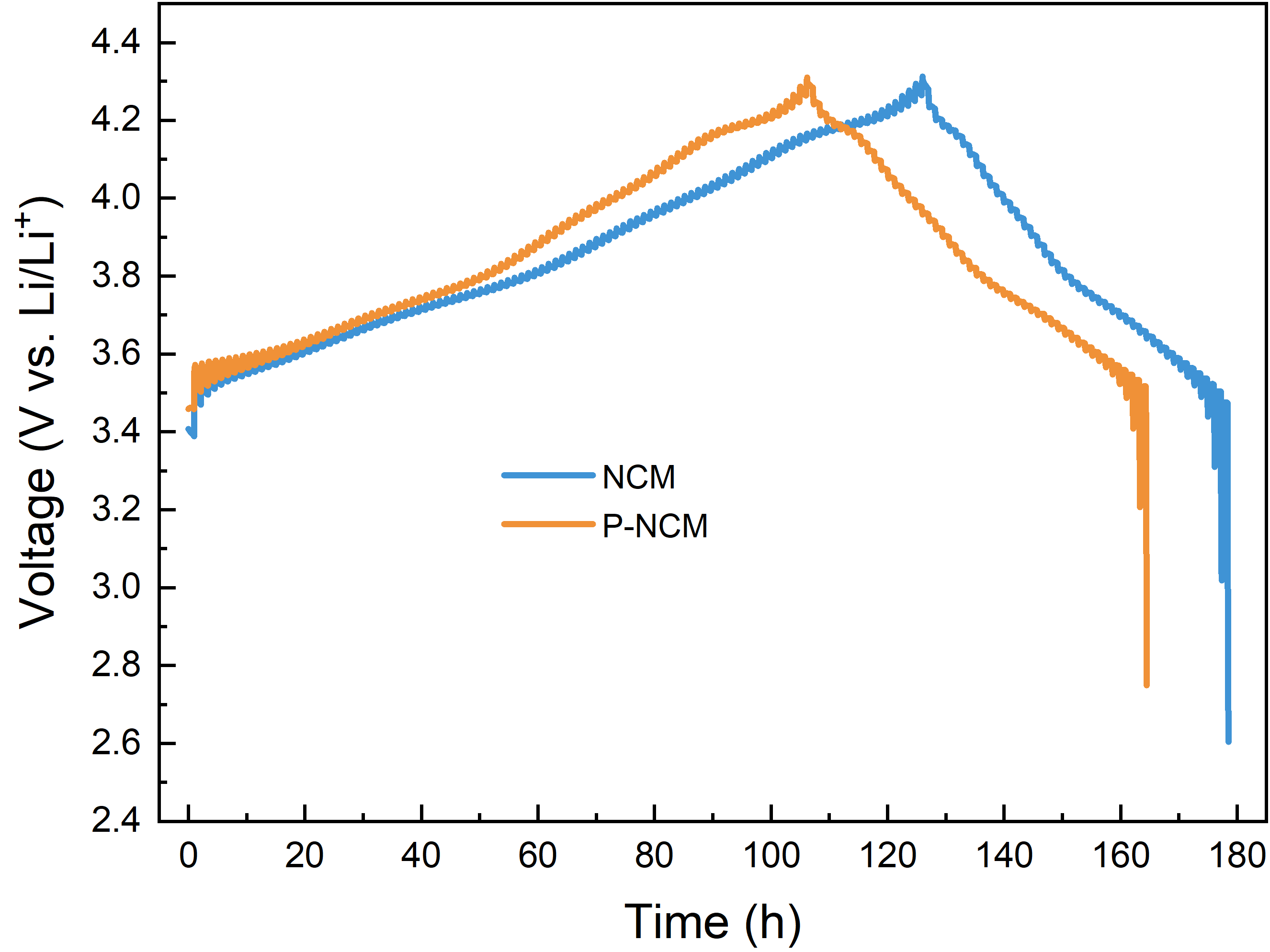


Figure S5. GITT curves of NCM and P-NCM.


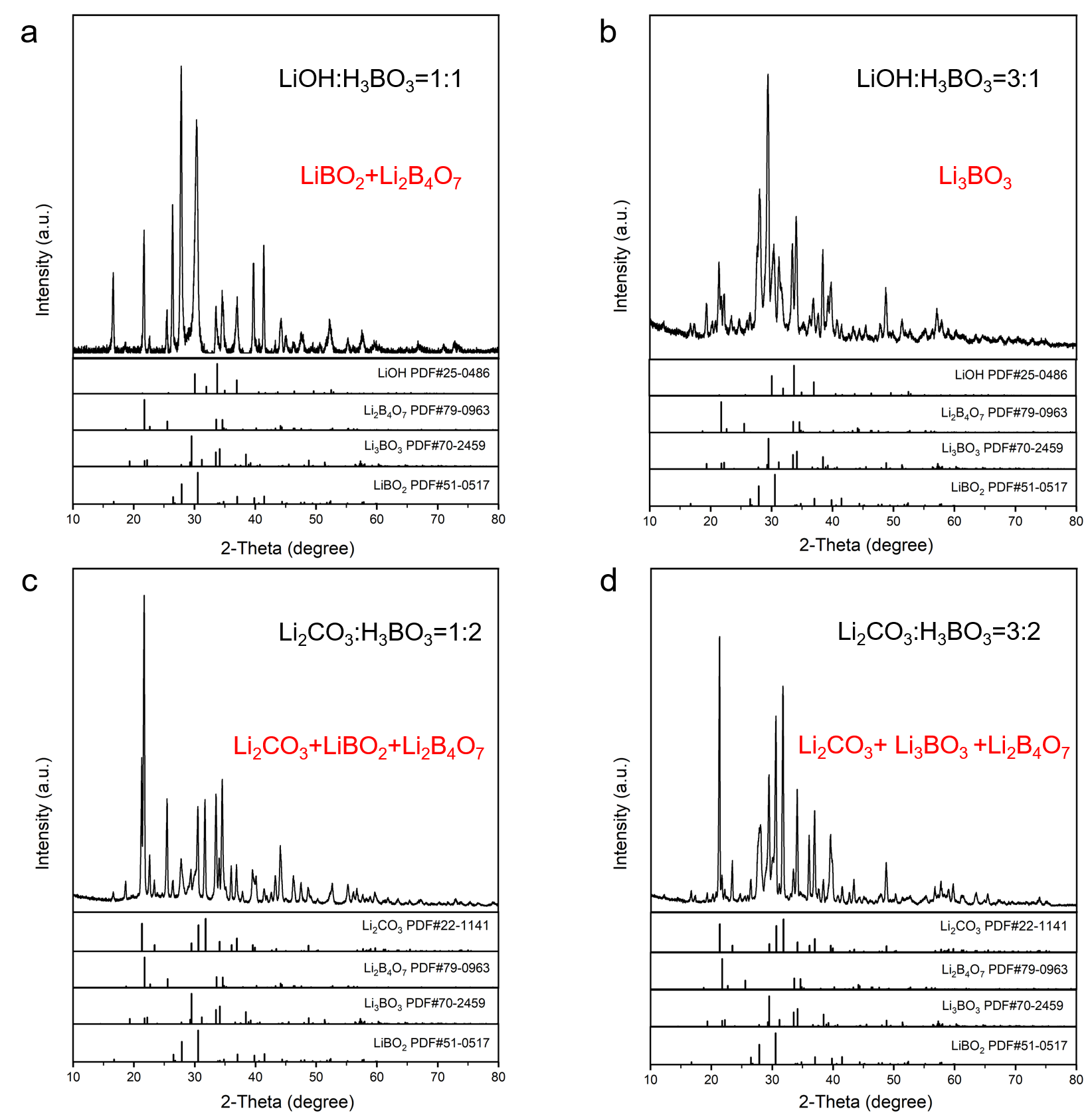


Figure S6. XRD patterns of matter calcinated at 500 ^o^C for 5 hours with raw materials of (a) LiOH:H_3_BO_3_=1:1, (b) LiOH:H_3_BO_3_=3:1, (c) Li_2_CO_3_:H_3_BO_3_=1:2 and (d) Li_2_CO_3_:H_3_BO_3_=3:2. Corresponding final products are highlighted in red.


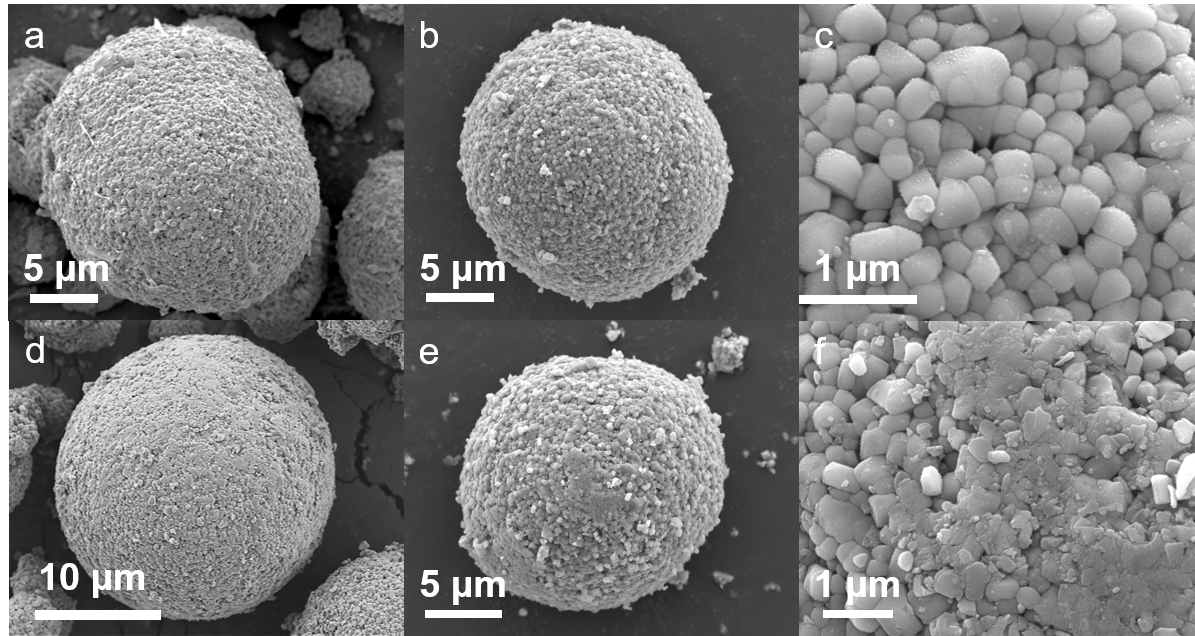


Figure S7. Morphologies of prepared cathode materials: (a) B0-NCM; (b-c) B1-NCM; (d) B5-NCM; (e-f) B10-NCM.


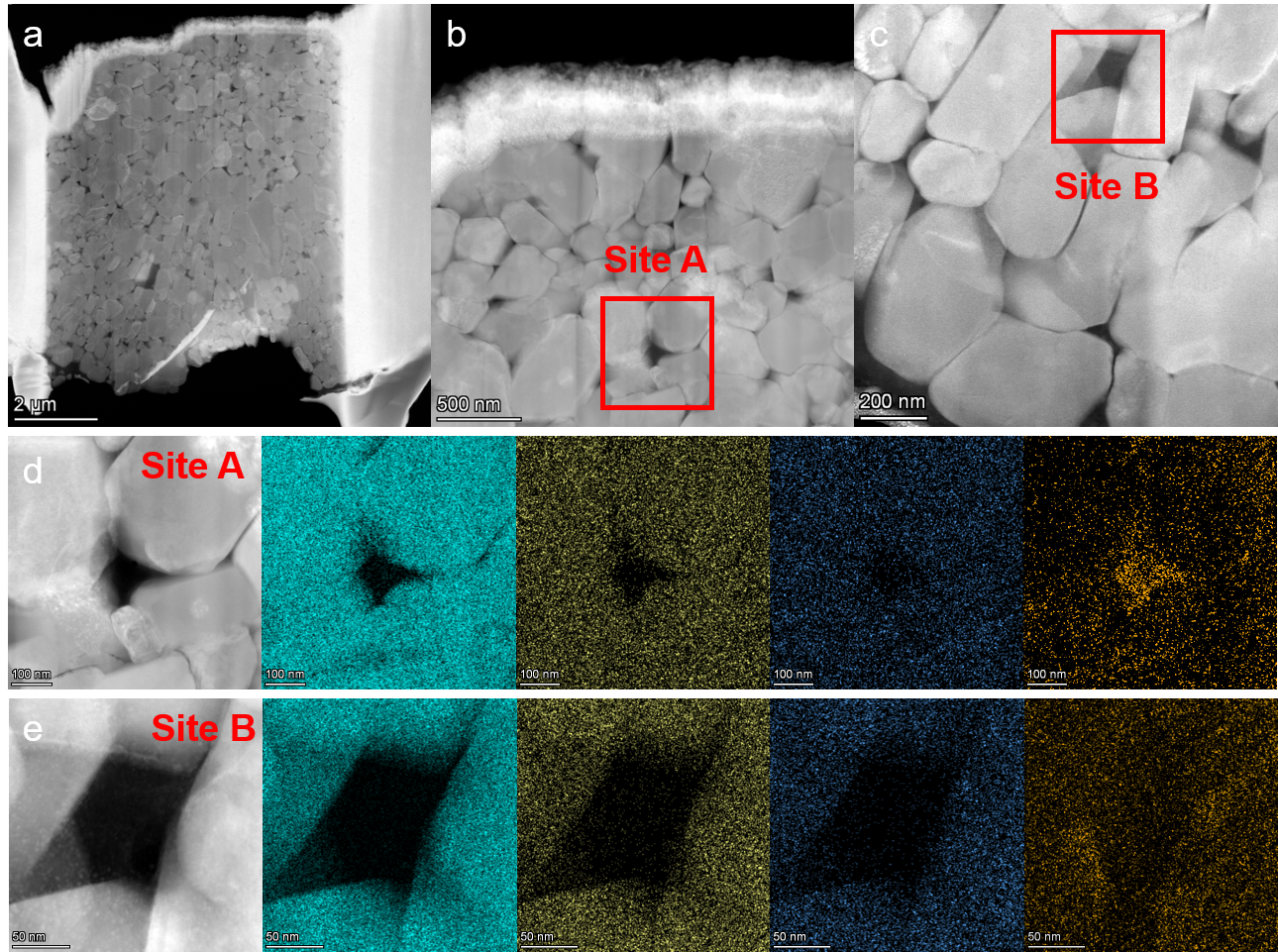


Figure S8. Cross-section diagrams of B5-NCM from HAADF-STEM (a-c) and corresponding EDS mapping results (d-e).


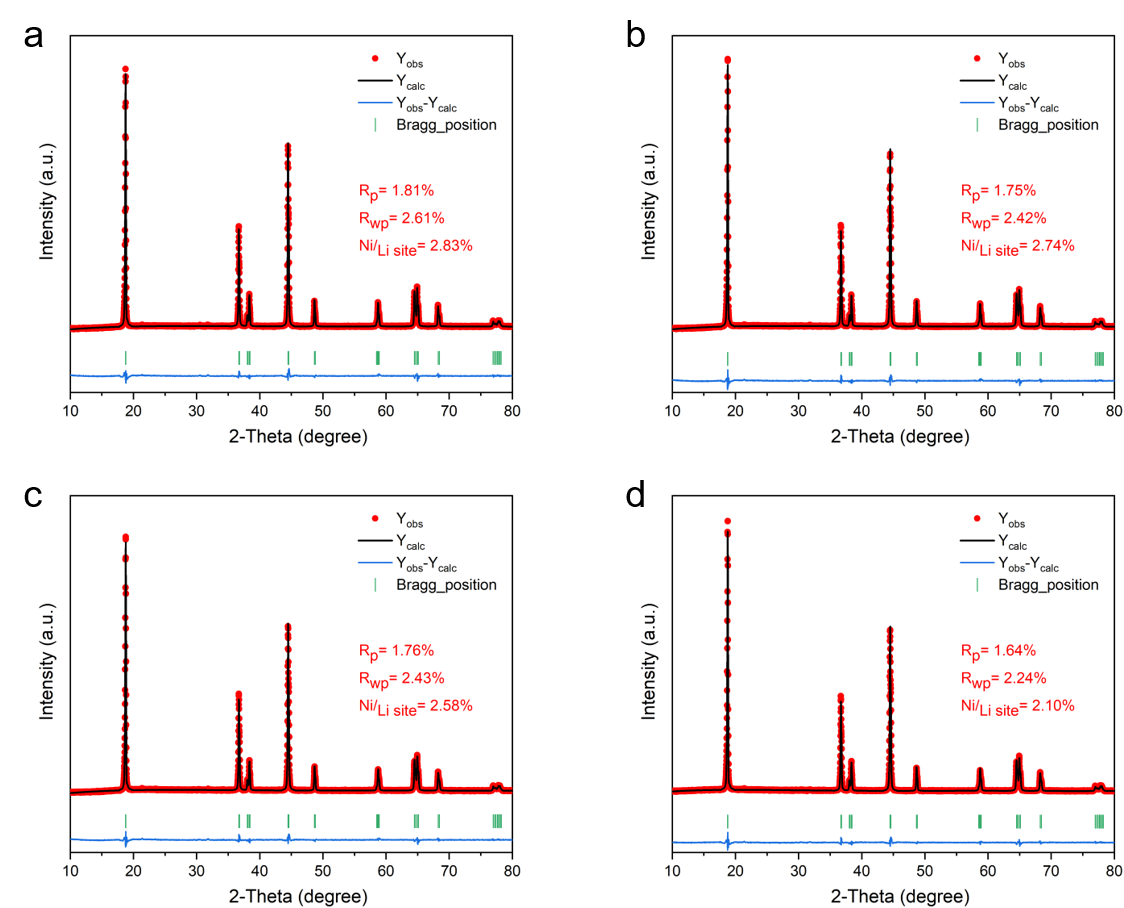


Figure S9. Rietveld refinement results of X-ray diffraction patterns for (d) B0-NCM; (e) B1-NCM; (f) B5-NCM; (g) B10-NCM.

Table S1 BET results of precursors and prepared cathode materials

| Samples | Hydroxide precursor | Oxide precursor | NCM | P-NCM | B0-NCM | B5-NCM |
| --- | --- | --- | --- | --- | --- | --- |
| BET surface area/ m^2^ g^-1^ | 4.6022 | 10.0163 | 6.1175 | 11.7099 | 10.4069 | 9.6071 |
| BJH Desorption cumulative volume of pores/ cm^3^ g^-1^ | 0.0181 | 0.0275 | 0.0228 | 0.0263 | 0.0343 | 0.0204 |
| BJH Adsorption average pore size/ nm | 13.9884 | 9.7104 | 13.2488 | 8.3852 | 11.79121 | 8.0191 |
| Median micropore width/ nm | 1.0376 | 1.0169 | 1.0404 | 1.0611 | 1.0391 | 1.0387 |

Table S2 Rietveld XRD refinement results of prepared cathode materials

| Samples | (003)/(104) | a/Å | c/Å | c/a | V/Å^3^ | I(LiO_2_) /Å | S(MO_2_) /Å | Ni/_Li site_ |
| --- | --- | --- | --- | --- | --- | --- | --- | --- |
| NCM | 1.55 | 2.86947(8) | 14.18676(37) | 4.94403 | 101.162(5) | 2.59145 | 2.13747 | 2.36% |
| P-NCM | 1.48 | 2.86932(8) | 14.18334(38) | 4.94310 | 101.127(5) | 2.56501 | 2.16277 | 2.74% |
| B0-NCM | 1.40 | 2.86989(8) | 14.18646(39) | 4.94321 | 101.190(5) | 2.56614 | 2.16268 | 2.83% |
| B1-NCM | 1.50 | 2.86914(8) | 14.18438(39) | 4.94377 | 101.122(5) | 2.56945 | 2.15867 | 2.74% |
| B5-NCM | 1.49 | 2.86907(8) | 14.18565(39) | 4.94434 | 101.126(5) | 2.57025 | 2.15830 | 2.58% |
| B10-NCM | 1.62 | 2.86900(8) | 14.18479(37) | 4.94416 | 101.115(5) | 2.59194 | 2.13632 | 2.10% |

Table S3 Summarization of electrochemical properties at 25 ^o^C

| Samples | 0.1C/mAh g^-1^ | 1C/mAh g^-1^ | 1C 100cycles/mAh g^-1^ | 1C 100 cycles Retention/% | 5C/mAh g^-1^ | 5C vs 0.1C |
| --- | --- | --- | --- | --- | --- | --- |
| NCM | 193.7 | 184.8 | 124.0 | 67.1% | 123.1 | 63.5% |
| P-NCM | 199.2 | 185.7 | 136.8 | 73.7% | 150.9 | 75.8% |
| B0-NCM | 200.2 | 183.3 | 134.7 | 73.5% | 154.6 | 77.2% |
| B1-NCM | 199.2 | 191.2 | 156.8 | 82.0% | 171.5 | 86.1% |
| B5-NCM | 205.5 | 195.1 | 159.4 | 81.7% | 175.6 | 85.5% |
| B10-NCM | 204.6 | 189.8 | 158.3 | 83.4% | 160.6 | 78.5% |

Table S4 EIS results of prepared cathode materials before and after cycles

| Samples | Before cycling | | | After cycling | | |
| --- | --- | --- | --- | --- | --- | --- |
|  | R_e_/Ω | R_ct_/Ω | R_e_/Ω | | R_sf_/Ω | R_ct_/Ω |
| NCM | 1.238 | 110.7 | 1.515 | | 50.56 | 846.5 |
| P-NCM | 1.303 | 77.2 | 1.676 | | 19.86 | 428. 2 |
| B0-NCM | 1.381 | 68.5 | 2.264 | | 22.78 | 483.9 |
| B1-NCM | 1.003 | 65.1 | 2.376 | | 14.75 | 325.9 |
| B5-NCM | 1.047 | 56.86 | 2.331 | | 27.31 | 268 |
| B10-NCM | 0.837 | 72.0 | 3.237 | | 15.45 | 503.5 |

Table S5 Results of average Li^+^ diffusion coefficient for prepared samples

| Samples | Average Li^+^ diffusion coefficient D (cm^2^ S^-1^) |
| --- | --- |
| NCM | 8.02*10^-11^ |
| P-NCM | 1.95*10^-10^ |
| B0-NCM | 1.69*10^-10^ |
| B5-NCM | 3.66*10^-10^ |

Table S6 Summarization of cycling performances at 60 ^o^C

| Samples | 0.1C  /mAh g^-1^ | 1C  /mAh g^-1^ | 1C 100 cycles  /mAh g^-1^ | 1C 100 cycles retention/% |
| --- | --- | --- | --- | --- |
| B0-NCM | 223.1 | 219.5 | 119.0 | 54.2% |
| B5-NCM | 223.0 | 219.4 | 154.9 | 70.6% |
